# ﻿Number and location of rDNA clusters in the superfamilies Tenthredinoidea and Cynipoidea (Hymenoptera): an update

**DOI:** 10.3897/compcytogen.18.142301

**Published:** 2024-12-04

**Authors:** Vladimir E. Gokhman, Valentina G. Kuznetsova, Boris A. Anokhin

**Affiliations:** 1 Russian Entomological Society, Moscow, Russia Russian Entomological Society Moscow Russia; 2 Zoological Institute, Russian Academy of Sciences, St. Petersburg, Russia Zoological Institute, Russian Academy of Sciences St. Petersburg Russia

**Keywords:** Apocrita, Argidae, chromosomes, Cynipidae, Hymenoptera, rDNA-FISH, Symphyta, Tenthredinidae

## Abstract

To identify nucleolus organizing regions (NORs), fluorescence *in situ* hybridization (FISH) with 18S rDNA probe was performed on chromosomes of *Tenthredocampestris* Linnaeus, 1758 (Tenthredinidae), *Argeciliaris* (Linnaeus, 1767) (Argidae) (n = 10 in both) and *Aulacideahieracii* (Bouché, 1834) (Cynipidae) (2n = 20). In all these species, a single pericentromeric rDNA cluster per haploid karyotype was detected. This number of NORs is confirmed as ancestral for the order Hymenoptera.

Hymenoptera represent one of the largest insect orders, with the approximate number of described species far exceeding 150,000 (Huber 2017). However, the overwhelming majority of this taxonomic diversity belongs to the suborder Apocrita, or higher Hymenoptera (see, e.g., Forbes et al. 2018), whereas the substantially less speciose Symphyta (= lower Hymenoptera) harbor less than nine thousand members (Huber 2017). Nevertheless, Symphyta include the least advanced Hymenoptera, and therefore studying these taxa is necessary to reconstruct ancestral character states for the order in general. Among other characteristics, this also applies to different karyotype features of Hymenoptera (Gokhman 2023a). For example, the ancestral nature of the canonical “insect-type” telomeric repeat in this order, TTAGG, was proven only when telomere structure of certain sawflies has been studied (Gokhman and Kuznetsova 2018; Lukhtanov and Pazhenkova 2023). However, other characteristics of symphytan chromosome sets, i.e., the number and location of clusters of 45S ribosomal DNA (rDNA), were studied in just a few members of the superfamily Tenthredinoidea, which belong to the families Tenthredinidae, Athaliidae and Diprionidae (Rousselet et al. 1999, 2000; Kuznetsova et al. 2001; Matsumoto et al. 2002). During these studies, different techniques for revealing 45S rDNA sites, which represent nucleolus organizing regions (NORs), i.e., AgNOR-banding, staining GC-enriched chromosome segments with chromomycin A_3_ (CMA_3_) and *in situ* hybridization with rDNA probes, including that using fluorescent dyes (FISH), were employed (Gokhman and Kuznetsova 2024). Nevertheless, the ancestral number and location of these rDNA clusters in the Symphyta remain ambiguous, since haploid karyotypes of two members of the genus *Tenthredo* Linnaeus, 1758, *T.velox* Fabricius, 1798 and *T.arcuata* Forster, 1771, were shown to have one and two 45S rDNA sites, respectively (Kuznetsova et al. 2001). Moreover, another species of the family Tenthredinidae, *Rhogogasterviridis* (Linnaeus, 1758), as well as *Diprionpini* (Linnaeus, 1758) and *Neodiprionabietis* (Harris, 1841) (Diprionidae) also have single 45S rDNA clusters (Rousselet et al. 1999, 2000; Kuznetsova et al. 2001), but the haploid karyotype of *Athaliarosae* (Linnaeus, 1758), which is now placed in a separate family Athaliidae (Wutke et al. 2024), carries four sites of that kind (Matsumoto et al. 2002).

Although much more is now known about the number and location of NORs in Apocrita (Gokhman and Kuznetsova 2024), reconstruction of the ancestral number and location of these parameters in this suborder is also far from straightforward. Specifically, both the number and location of these sites vary substantially across parasitoids as well as across aculeate Hymenoptera. Whilst the only 45S rDNA cluster per haploid karyotype is a widespread condition in Apocrita (see, e.g., Gokhman et al. 2024), chromosome sets of these insects can harbor up to six and even 15 NORs in parasitic wasps and Aculeata, respectively (Gokhman and Kuznetsova 2024). FISH also visualized single 45S rDNA clusters in all studied members of the superfamily Cynipoidea, i.e., in *Diplolepisrosae* (Linnaeus, 1758) (Cynipidae) (Gokhman et al. 2014) as well as in four species of the family Figitidae (Gokhman et al. 2016). In addition, karyotypes of three other members of Cynipidae, *Aulacideahieracii* (Bouché, 1834), *Isocolusjaceae* (Schenck, 1863) and *I.scabiosae* (Giraud, 1859), have been recently examined using CMA_3_ (Gokhman 2021). Although single CMA_3_-positive sites per haploid chromosome set were detected in all these species, these results could be corroborated using FISH, since CMA_3_-positive chromosome segments do not always represent NORs (see, e.g., Gromicho et al. 2005; Gokhman et al. 2017). We have therefore undertaken the present study to further identify the number and location of rDNA clusters in the superfamilies Tenthredinoidea and Cynipoidea. The results of this study are given below.

## ﻿Material and methods

### ﻿Origin of insects

Adult sawflies and galls containing immature stages of *A.hieracii* were collected in 2022 by the first author near Ozhigovo, Russia (about 60 km SW Moscow) as well as by M.I. Nikelshparg (Saratov State University, Saratov, Russia) near the city of Saratov, Russia (about 730 km SE Moscow), respectively. All sawflies were identified by S.A. Basov (Zoological Institute, Russian Academy of Sciences, St. Petersburg, Russia).

### ﻿Preparation of chromosomes

Chromosome preparations were obtained according to the guidelines provided by Naito (1982) and Imai et al. (1988) with a few modifications. Adult female sawflies were dissected in small Petri dishes in distilled water, unfertilized mature eggs were extracted from their bodies, placed into the dishes on a filter paper soaked with distilled water, and then incubated for 3–4 days at room temperature (RT). Haploid embryos and cerebral ganglia were extracted from the eggs and gall wasp prepupae, respectively, and dissected in 0.5% hypotonic sodium citrate solution containing 0.005% colchicine. The embryos and ganglia were then transferred to a fresh portion of hypotonic solution and incubated for about 30 min at room temperature. The material was transferred onto a pre-cleaned microscope slide using a Pasteur pipette and then gently flushed with Fixative I (glacial acetic acid: absolute ethanol: distilled water 3:3:4). The tissues were disrupted using dissecting needles in an additional drop of Fixative I. A drop of Fixative II (glacial acetic acid: absolute ethanol 1:1) was applied to the center of the area, and the more aqueous phase was blotted off the edges of the slide. The slides were then dried and stored at RT for a few weeks.

### ﻿FISH procedure

Genomic DNA from a male *Pyrrhocorisapterus* (Linnaeus, 1758) (Hemiptera, Heteroptera, Pyrrhocoridae) was isolated using CTAB extraction method. FISH with the 18S rRNA gene probe was carried out on chromosomes of all studied species. The target 18S rRNA gene was PCR amplified (see Grozeva et al. 2011 for the table of primers) from the genomic DNA of *P.apterus*, and labeled by PCR with biotin.

*In situ* hybridization was performed as described by Schwarzacher and Heslop-Harrison (2000) with modifications. Chromosome preparations were dehydrated through 70, 80 and 96% ethanol at RT and treated with 100 µg/ml RNAse A (Sigma) for 30 min at 37 °C in a humid chamber; washed three times in 2× SSC (5 min each) at RT; dehydrated through 70, 80 and 96% ethanol at RT; incubated in 5 mg/ml pepsin in 0.01 N HCl for 10 min at 37 °C; washed sequentially in PBS, in PBS containing 0.05 M MgCl_2_ for 5 min each, in 1% PFA in PBS containing 0.05 M MgCl_2_ for 15 min, in PBS for 5 min, in PBS containing 0.05 M MgCl_2_ for 5 min at RT each; dehydrated through 70, 80 and 96% ethanol at RT and finally, dried. After pretreatment, 6.5 µl hybridization mixture containing about 100 ng of labeled probe, 50% formamide, 2× SSC, 10% (w/v) dextran sulfate, 1% (w/v) Tween 20 and 10 µg salmon-sperm DNA was added on preparations. Slides were mounted using glass coverslips and rubber cement. The slides were denatured for 5 min at 75 °C. The chromosome slides were then incubated for 42–44 h at 37 °C. Following hybridization, the slides were washed in 2× SSC for 3 min at 45 °C, then in 50% formamide in 2× SSC for 10 min at 45 °C, two times in 2× SSC (10 min each) at 45 °C, blocked in 4× SSC containing 1.5% (w/v) BSA and 0.1% Tween 20 for 30 min at 37 °C in a humid chamber. 18S rRNA gene probe was detected with 5 µg/ml Avidin-FITC (Invitrogen). Detection was performed in 4× SSC containing 1.5% BSA and 0.1% Tween 20 for 1 h at 37 °C. Slides were washed three times in 4× SSC containing 0.02% Tween 20 (10 min each) at 45 °C and dehydrated through 70, 80 and 96% ethanol at RT. Chromosomes were mounted in an antifade medium (ProLong Gold antifade reagent with DAPI, Invitrogen) and covered with a glass coverslip.

### ﻿Image acquisition and analysis

Metaphase plates and interphase nuclei were analyzed under a Leica DM 6000B microscope with a 100× objective. Fluorescence images were taken with a Leica DFC 345 FX camera using Leica Application Suite 4.5.0 software with an Image Overlay module. To prepare illustrations, the resulting images were arranged and enhanced using GIMP 2.10. Chromosomes were classified according to the guidelines provided by Levan et al. (1964).

## ﻿Results

**Superfamily Tenthredinoidea**

**Family Tenthredinidae**

***Tenthredocampestris* Linnaeus, 1758**

**Figure 1.**
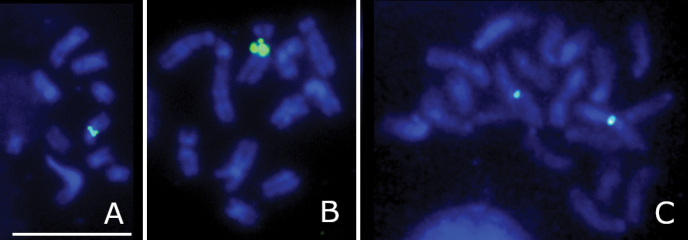
FISH with 18S rDNA probe on Hymenoptera chromosomes. *Tenthredocampestris*, n = 10 (**A**), *Argeciliata*, n = 10 (**B**), *Aulacideahieracii*, 2n = 20 (**C**). Probe signals are indicated in green. Scale bar: 10 µm.

A detailed description of the karyotype of this species can be found in Gokhman (2024). The haploid set of *T.campestris* includes 10 biarmed chromosomes. In the karyotype of this species, the first chromosome is about 1.3 times longer than the second one, which is, in turn, also 1.3 times longer than the third chromosome. The remaining chromosomes more or less gradually decrease in size. The haploid karyotype of *T.campestris* harbors the only pericentromeric rDNA cluster on a particular medium-sized chromosome (Fig. 1A).

**Family Argidae**

***Argeciliaris* (Linnaeus, 1767)**

A detailed karyotypic description of this species is given by Gokhman (2023b). The haploid set of *A.ciliaris* harbors 10 biarmed chromosomes. The first chromosome is about 1.8 times longer than the second one; the remaining chromosomes gradually decrease in size. On some metaphase plates, the second metacentric bears an obvious pericentromeric secondary constriction on its longer arm (see Gokhman 2023b). It is therefore not surprising that the only 18S rDNA site revealed in this species by FISH is apparently co-localized with this constriction (Fig. 1B).

**Superfamily Cynipoidea**

**Family Cynipidae**

***Aulacideahieracii* (Bouché, 1834)**

A detailed description of the karyotype of this species, including results of CMA_3_ staining can be found in Gokhman (2021). The diploid set of *A.hieracii* includes 20 chromosomes, which gradually decrease in size, with a single pericentromeric CMA_3_-positive band located on the largest metacentric. FISH with the 18S rDNA probe revealed the only paired site in place of the CMA_3_-positive band (see Gokhman 2021), thus suggesting that both used techniques visualized the same rDNA cluster (Fig. 1C).

## ﻿Discussion

Haploid karyotypes of both studied members of Tenthredinoidea, *T.campestris* (Tenthredinidae) and *A.ciliaris* (Argidae), harbor single rDNA clusters. Moreover, this character state predominates within Tenthredinoidea (see above), and this therefore suggests that the single rDNA site per haploid chromosome set is ancestral at least for the whole superfamily. This is further corroborated by the fact that *A.ciliaris* remains the only examined member of the Argidae + Pergidae clade, a sister one to the remaining Tenthredinoidea except Blasticotomidae (Wutke et al. 2024), the most basal family which chromosomes are totally unknown. If this is true, then higher numbers of rDNA clusters found in *Tenthredoarcuata* and *Athaliarosae* (Kuznetsova et al. 2001; Matsumoto et al. 2002) apparently represent apomorphic conditions. Anyway, studying these clusters in other superfamilies of the Symphyta could either confirm or reject this assumption.

In turn, Tenthredinoidea also represent a sister clade to all other Hymenoptera, except for the family Xyelidae (Wutke et al. 2024), in which the structure and location of rDNA sites are still unknown (see Gokhman 2023a). Nevertheless, a single 45S rDNA cluster per haploid karyotype can be found in the majority of parasitoid Hymenoptera including all known Cynipoidea (Gokhman et al. 2014, 2016; Gokhman 2022; present paper). Furthermore, the same character state is considered ancestral for Aculeata, despite wide variation in the number and location of these clusters in aculeate Hymenoptera (Menezes et al. 2021). Taken together, all this information suggests that a single rDNA site per haploid genome is also ancestral for the whole order Hymenoptera. Again, this does not seem surprising, since a similar pattern is apparently characteristic of the class Insecta in general (Gokhman and Kuznetsova 2024).
